# The impact of podoconiosis, lymphatic filariasis, and leprosy on disability and mental well-being: A systematic review

**DOI:** 10.1371/journal.pntd.0009492

**Published:** 2021-07-08

**Authors:** Oumer Ali, Asrat Mengiste, Maya Semrau, Abraham Tesfaye, Abebaw Fekadu, Gail Davey

**Affiliations:** 1 CDT-Africa, College of Health Sciences, Addis Ababa University, Addis Ababa, Ethiopia; 2 Centre for Global Health Research, Brighton and Sussex Medical School, University of Sussex, Brighton, United Kingdom; 3 School of Public Health, College of Health Sciences, Addis Ababa University, Addis Ababa, Ethiopia; RTI International, UNITED STATES

## Abstract

Leprosy, podoconiosis, and lymphatic filariasis (LF) are among the priority neglected tropical diseases (NTDs) in Ethiopia. The disability, psychosocial, and mental health status of people affected by these NTDs are still overlooked in global NTD discourse. The objective of this systematic review was to synthesize the existing evidence describing the disability, psychosocial, and mental health status of people affected by leprosy, podoconiosis, and LF prior to developing a holistic physical and psychosocial care package for these individuals. We searched papers reporting on disability, psychosocial, and mental health status linked to these 3 NTDs. The protocol was registered in PROSPERO with registration number CRD42019128400. Peer-reviewed articles were searched and extracted from Medline, PsycINFO, Global Health, and Embase. Articles published in English, irrespective of the year of publication, using a quantitative study methodology, were included. Abstracts and full texts were reviewed by 2 reviewers. Data were extracted and narratively summarized, as the studies were heterogeneous and used different outcome measures. Out of 1,318 titles/abstracts screened and 59 full text studies reviewed, 24 fulfilled the inclusion criteria. Fourteen studies provided evidence of the disability associated with leprosy, podoconiosis, or LF. Ten studies provided evidence on the association between the 3 NTDs and mental health or psychosocial outcomes. The prevalence of grade 2 disability varied from 3.9% to 86%. The most commonly reported mental health impacts were depression and mental distress. A high burden of mental illness was reported, varying from 12.6% to 71.7%; the suicidal ideation was also high (18.5%). In conclusion, disability and poor psychosocial and mental health status are associated with leprosy, podoconiosis, and LF. For optimum management of these NTDs, holistic care including both physical and psychosocial interventions is vital.

## Author summary

Podoconiosis, lymphatic filariasis (LF), and leprosy are neglected tropical diseases (NTDs) that are commonly found among disadvantaged rural and some poor urban communities. These diseases lead to disabilities of the leg, which may result in reduced productivity. The longer the duration of the disease, the more severe the disability. People affected by these diseases also commonly experience stigma and discrimination, which, in turn, affect health-seeking behavior. Due to this disability, stigma, and discrimination, people affected by these 3 NTDs have a poor quality of life. We also found a range of mental health problems related to these 3 NTDs. Among these, the most common were mental distress and depression. As all 3 NTDs are associated with poor physical, psychosocial, and mental health outcomes, management should be holistic, incorporating physical, psychosocial, and mental health interventions. Following this review, we are working on the development of a holistic integrated care package for people affected by leprosy, podoconiosis, and LF in northwestern Ethiopia.

## Introduction

Neglected tropical diseases (NTDs) are a group of chronic, disabling, and disfiguring conditions that occur most commonly in settings of extreme poverty, especially among the rural poor and some underprivileged urban populations in low- and middle-income countries. Despite the significant disease burden they impose, these conditions were largely ignored within the global health field until grouped as “NTDs” in 2000. Social stigma, prejudice, marginalization, and the extreme poverty of afflicted populations are among the factors contributing to the neglect of these diseases [1]. The 3 NTDs addressed in this review are leprosy, podoconiosis, and lymphatic filariasis (LF). These 3 conditions were selected because they are priority NTDs in Ethiopia and because this review was carried out as part of an implementation research study on integrating care for these 3 conditions (the EnDPoINT program).

Leprosy is a chronic infectious disease caused by the bacterium *Mycobacterium leprae*. The disease affects the skin, peripheral nerves, limbs, and eyes; it can cause severe disability and may take 20 years or more to develop after onset of infection [2]. According to the 2019 World Health Organization (WHO) report, at the end of the 2019 reporting year, there were 202,185 new cases; among these, 177,175 were on multidrug therapy (MDT). In 2019, Brazil, India, and Indonesia reported more than 10,000 cases (80% of all cases). In the same calendar year, 13 countries, including Ethiopia, reported 1,000 to 10,000 cases. About 99 countries reported fewer than 1,000 cases. Among the reported cases, 7.4% were among children under 15 years of age. Moreover, from the registered new cases in 2019, there were 5.3% grade II disabilities [3]. In Ethiopia, in 2015, a total of 3,758 new leprosy cases were registered, and, among these, 12.8% were children. A total of 10.2% of new cases of leprosy had grade II disability at diagnosis [4].

Podoconiosis primarily affects barefoot underprivileged farmers in areas with red volcanic soil as a consequence of long-term exposure to this soil. The disease results from interaction between genetic susceptibility and the environment [5–6]. Clinical sequelae include progressive bilateral leg swelling, resulting in disability; the disease is also complicated by acute adenolymphangitis, which results in reduced productivity [7]. A recent systematic search of available data reported podoconiosis to be present in 17 countries: In Africa, there was complete consensus of its presence in 6 countries (Cameroon, Ethiopia, Kenya, Rwanda, Tanzania, and Uganda) and weaker evidence of presence in 6 other countries; in Asia, there was evidence of presence in 2 countries (India and Indonesia); and in Latin America, there was moderate evidence for the presence of podoconiosis in 3 countries (Brazil, Ecuador, and Mexico) [8]. In Ethiopia, podoconiosis is endemic in 345 of the 775 surveyed districts, and it is estimated to affect approximately 1.5 million people, with a further 34.9 million people at risk of the condition [9].

LF is an NTD that is prevalent in 73 tropical and subtropical countries. LF is caused by 3 species of filarial worms—*Wuchereria bancrofti*, *Brugia malayi*, and *Brugia timori*—and is transmitted by multiple species of mosquitoes. There are varied clinical manifestations; the most common are hydrocele and chronic lymphedema of the legs or arms [10]. Just as for podoconiosis, one of the most disabling complications of the disease is acute adenolymphangitis. LF is reported within the tropics, including in East/Southeast Asia, Oceania, Africa, and South America. Globally, there are an estimated 67.88 million LF cases, including 36.45 million microfilaria carriers, 19.43 million hydrocele cases, and 16.68 million lymphedema cases [11]. LF is endemic in 70 of the 658 surveyed districts, with the estimated population at risk of the condition being 5.9 million [12].

Despite the high prevalence and apparent physical and psychosocial burdens imposed by leprosy, podoconiosis, and LF, there is a gap in the literature in identifying specific disability, psychosocial, and mental health outcomes in a systematic way.

According to WHO, disability, psychosocial, and mental health conditions are defined as follows:

“Disability is defined as: problems in body functions or structure such as deviations or loss, difficulties individuals may have in executing activities, and problems an individual may experience in involvement in life situations [13].”

“Psychological distress comprises the worry, fears, sadness and insecurity often experienced by people with an NTD and the associated stigma. It can result in reduced social functioning and self-isolation [14].”

“Mental health conditions are characterized by changes in thoughts, perceptions, emotions or behaviour that affect relationships and the ability to perform expected social roles and can cause significant functional impairment. Some examples include depression, anxiety, harmful use of alcohol and other psychoactive substances [14].”

### Research questions

What are the prevalent disabilities, psychosocial, and mental health effects to be considered in order to develop a holistic physical and psychosocial care package for those affected by leprosy, podoconiosis, and LF?

The objective was to determine the disability outcomes secondary to LF, podoconiosis, and leprosy, as well as the psychosocial–mental health outcomes secondary to these conditions. This review was conducted prior to developing a holistic physical and psychosocial care package for individuals affected with podoconiosis, LF, or leprosy.

Although the 3 diseases have different etiologies and pathogenesis, all lead to leg deformity, which profoundly affects productivity (although LF and leprosy affect other parts of the body too). Due to this, potential integration of care for these diseases at the primary healthcare level is a possibility.

## Methods

### Literature search

#### Eligibility criteria

Eligible studies were those addressing the NTDs podoconiosis, LF, or leprosy, or any combination of these. The outcome measures focused upon for these diseases were disability and psychosocial or mental health outcomes. We only included articles with outcomes measured using standard tools. Only studies published in English, for which the full text was available, were included. There was no restriction on publication year. Studies that were not published in peer-reviewed scientific journals, or were either purely qualitative studies or animal studies, were excluded.

The protocol is available at the National Institute for Health Research PROSPERO International prospective register of systematic reviews (identifier: CRD42019128400) (see S1 Text).

#### Searches

From July to August 2019, studies were identified by systematic search of 4 electronic databases: Medline, Global Health, PsycINFO, and Embase. Additionally, we included one article published by one of the coauthors that had yet to be indexed in any of the literature databases searched. We used search terms for NTDs; for disability, psychosocial, and mental health outcomes; and countries endemic for at least one of the 3 NTDs.

The following search terms were used in all 4 databases, where we searched the main domains and their synonyms. The search terms for NTDs were “podoconiosis” OR “lymphatic filariasis” OR “leprosy” OR “elephantiasis” OR “elephantiasis, filarial.” The search terms for disability, psychosocial, and mental health outcomes included the following: “disability” OR “functioning” OR “mental distress” OR “depression” OR “alcohol abuse” OR “substance abuse” OR “psychosocial” OR “anxiety disorder” OR “common mental disorder” OR “mood disorder” OR “distress” OR “major depression” OR “depressive disorder” OR “alcohol” OR “substance” OR “anxiety” OR “mental disorder.” The list of endemic countries for leprosy and lymphatic filariasis were taken from recent WHO reports [15–16] and for podoconiosis from a recent systematic review [17]; all of these countries were also included as search terms.

#### Study selection

**Fig 1:**
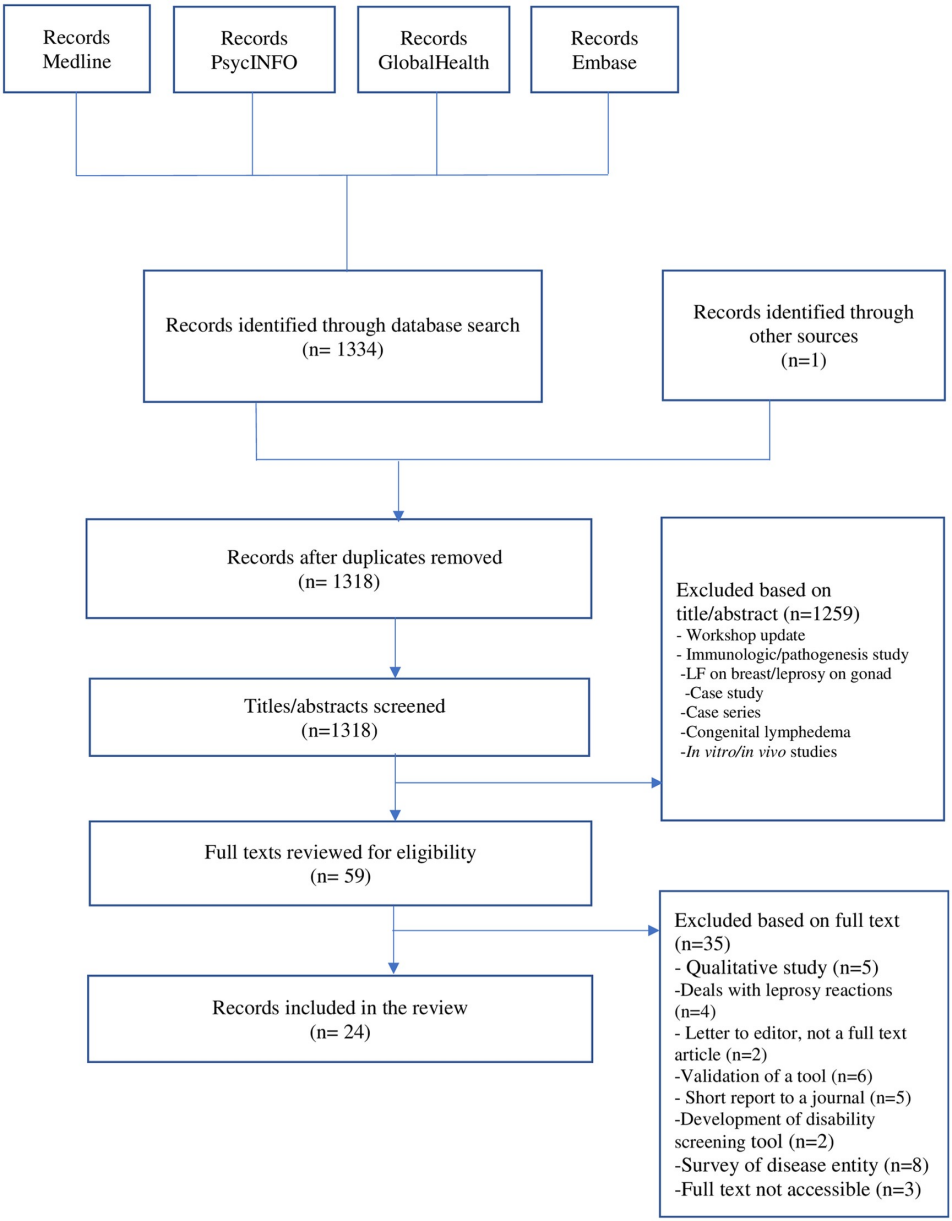
Flow diagram describing the study selection process. LF, lymphatic filariasis.

Studies that were identified through the database searches underwent a 2-stage screening process. First, 2 reviewers (OA and AM) screened the titles and abstracts using Endnote reference manager to remove duplicates and identify eligible articles based on the inclusion and exclusion criteria. Following the selection of articles through the title and abstract review, the full text articles were reviewed by the same 2 reviewers. After the 2 reviewers independently screened all articles, they met to achieve consensus on inclusion/exclusion of each article. The details are depicted in Fig 1.

#### Data extraction and analysis

The following data were extracted from studies that fulfilled inclusion and exclusion criteria: the disability, psychosocial, and mental health outcomes due to 3 NTDs; the outcome measures; and the number of studies conducted using the tool or the outcome measure (see Table 1).

**Table 1:** Profile of the disability, psychosocial, and mental health outcomes found among those affected by leprosy, podoconiosis, and LF and the outcome measures used in the studies.

	Leprosy	Tool used (no. of studies)	Podoconiosis	Tool used (no. of studies)	LF	Tool used (no. of studies)	Total
**Disabilities**	Hand and feet deformity	EHF scale (3)					
	Disability	WHO tool (6)	Disability	WHODAS (1)	Disability	8D5L survey tool (1)	
	Activity limitations	SALSA (4)			Limitation of activities	Functional measure (1)	
	Disability-adjusted life work years	DALY (1)					
	Social participation	P scale (2)					
	Total	16		1	1	2	20
**Psychosocial**	Quality of life	WHO-QoL brief (2)	Quality of life	WHO-QoL brief (1)	Quality of life	SF-36 (1)	
	-	-	-	-	-	-	
	Total	1		1		2	4
**Mental health**	Depression	CES-D (1)	Depression	PHQ-9 (2)	Depression	CIDI (1)	
	Mental distress	SRQ-20 (2)	Mental distress	Kessler-10 (1)	Current mental health status	SRQ-20 (1)	
	Psychiatric disorders	MINI-plus (1)			Mental health conditions	GHQ-30 (1)	
	Mental health conditions	GHQ-30 (1)					
	Total	5		3		3	11

8D5L, 8 domain/5-level; CIDI, Composite International Diagnostic Interview; CES-D, Center for Epidemiological Studies-Depression; DALY, disability-adjusted life year; EHF, Eye, Hand, and Feet; GHQ-30, General Health Questionnaire-30; LF, lymphatic filariasis; MINI-plus, Mini-International Neuropsychiatric Interview-Plus; PHQ-9, Patient Health Questionnaire-9; QoL, quality of life; SALSA, Screening of Activity Limitation and Safety Awareness; SF-36, Short-Form 36; SRQ-20, Self-Reporting Questionnaire-20; WHO, World Health Organization; WHODAS, WHO Disability Assessment Schedule.

The quality assessment mechanism was adapted from the Evidence for Policy and Practice Information and Co-ordinating Centre and included 6 quality criteria—aims clearly stated, design appropriate to the stated objectives, justification given for sample size, evidence provided of reliability or validity of measures used, statistics accurately reported, and sample selection relatively unbiased (i.e., where steps such as random sampling had been taken).

## Results

We identified 1,334 articles: 1,247 articles from Medline, 47 articles from PsycINFO, 14 articles from Global Health, and 26 from Embase. After removing duplicates, 1,318 articles were reviewed, of which 59 were included based on the title–abstract review. The full texts of these 59 articles were then reviewed, of which 23 were accepted for inclusion. One recent article known to the authors was retrieved through Google Scholar. The full text was unavailable for 3 articles.

### Description of included studies

The 24 studies included in this review were published between 1980 and 2019. Several study designs were used, including cross-sectional, case–control, and prospective studies. The majority of studies employed a cross-sectional study design. A significant number of the studies (*n* = 10) were conducted in Africa, and about half of these (*n* = 6) were conducted in Ethiopia. Besides Africa, most studies were conducted in Asia (*n* = 8), and half of these (*n* = 4) were from India. Six studies were conducted in Latin America. Among the articles included for review, 16 dealt with leprosy, 4 with podoconiosis, and another 4 with LF.

The aims were clearly stated in all articles included in this review, the design was appropriate to the stated objectives in 22/24 papers (92%), proper justification was given for sample size in 12/24 (50%), evidence for reliability or validity of measures was provided in 17/24 (71%), statistics were accurately reported in 20/24 (83%), and sample selection was relatively unbiased in 15/24 (63%).

The description of outcome measures used in the articles selected for this review are included in Table 2.

**Table 2:** Description of outcome measures.

Name of scale	Number of items	What the scale measures	Condition the tool has been used for	How it is scored	Source reference
WHO-DG	3	This scale measures disability based on WHO definition of disability in leprosy	To measure leprosy-related disability	Grade 0 implies no disability, grade 1 implies patients only have loss of sensation, and grade 2 indicates there is visible deformity.	[13]
EHF	12	The EHF score is the sum of disability of both eyes, and both hands and feet in leprosy patients	To measure leprosy-related disability	The scores range from 0 to 12. A higher score is associated with high grade of disability.	[18]
DAWLY	NA	This estimates the number of productive years lost due to the disability and can be termed disability-adjusted productive work years lost or DAWLY	Measures productivity years lost due to disability	The number of lost productive years due to disability recorded.	[19]
WHODAS II	12	The WHODAS II tool was developed by WHO to measure general disability.	To measure disability in podoconiosis cases and controls	Questions relate to concentration, physical activities of daily life, and social interactions over the last 30 days. Higher scores are related to higher disability.	[20]
SALSA	20	The SALSA scale measures activity limitations and risk awareness in patients who have or have had a disease with peripheral neuropathy, as in leprosy. The scale includes assessment of the eyes, hands (skills and labor), feet (mobility), and self-care.	To measure activity limitation related to leprosy	SALSA scores range from 10 to 80, with 10–24 allocated to patients without significant limitations; 25–39 for mild limitations; and 40–49, 50–59, and 60–80 for moderate, severe, and very severe limitations, respectively.	[21]
Participation scale	18	The Participation scale is composed of 18 items, which measures activities of social participation.	To measure social participation restriction in leprosy patients	Scores range from 0 to 90. The higher the score, the more severe the participation restriction. The levels of restriction are classified as follows: no restriction (0 to 12), mild restriction (13 to 22), moderate restriction (23 to 32), severe restriction (33 to 52), and extreme restriction (53 to 90).	[22]
WHOQOL-BREF	26	The WHOQOL-BREF was developed by WHO as a shortened version of the quality of life measure WHOQOL-100, and it assesses quality of life.	To measure quality of life in podoconiosis and leprosy cases	The WHOQOL-BREF uses a 5-point scale for each answer, and these are scored positively, with higher values indicating a higher quality of life.	[23]
PHQ-9	9	The PHQ-9 is used to screen depression.	To measure depression in podoconiosis cases	The 4 response categories refer to the amount of time the symptom was present from “not at all” (0) to “nearly every day” (3). Higher scores are associated with more severe forms of depression. Those who screen positive (with a score of 5 and above) can be further interviewed using the CIDI.	[24]
CIDI	9	A fully structured nonclinical interview designed for use in general population surveys or other study designs where clinical ratings are not practical. It can also be used for clinical purposes and is designed to assess mental disorders.	To assess depression in LF cases	Symptoms have been present during the same 2-week period, and at least one of the symptoms is either depressed mood or loss of interest or pleasure. Each symptom assessed as “change from previous functioning” corresponding to each symptom (e.g., “more than usual” and “less than usual”).	[25]
CES-D	20	CES-D scale is a brief self-report scale, which was developed to measure self-reported symptoms associated with depression experienced in the past week.	To assess depression in leprosy cases	It contains 20 items with 0–3 subitems covering the major components of depression. Higher scores indicate more severe depression.	[26]
Kessler-10	10	The Kessler-10 scale is a 10-item screening tool that measures the likelihood of some form of common mental disorder, such as depression or anxiety.	To measure podoconiosis-related mental distress	There are 10 questions, each scored out of 5. Higher mental distress scores indicate an increased probability of having depression or an anxiety disorder.	[27]
MINI-Plus	10	MINI-Plus is a short, structured diagnostic psychiatric interview for Diagnostic and Statistical Manual of Mental Disorders, fourth edition (DSM-IV) disorders. It is a short and accurate measure designed for clinical trials, epidemiologic research, and outcome tracking in nonresearch settings.	To assess psychiatric diagnosis in leprosy cases	The questionnaire comprises 10 Likert-type statements scored from 0 = do not agree at all, to 3 = agree fully.	[28]
SRQ-20	20	SRQ was developed by WHO to screen for psychiatric disturbance in primary healthcare settings in low-income countries.	To assess mental distress in leprosy cases and other dermatologic illnesses	It can be used as a first-stage screening instrument for the second-stage clinical interview. The questions ask about features of common mental disorders, particularly anxiety and depression. If the participant thinks the question applies to him/her, they will answer yes, and otherwise, the answer will be no.	[29]
GHQ-30	30	GHQ-30 is a measure of the current mental health status of individuals.	To measure the mental health status of LF and leprosy patients and controls	The GHQ-30 has 4 response categories for each of the 30 questions: better than usual, same as usual, less than usual, and much less than usual. The scoring method is categorized into a dichotomous response (“0” for the first 2 options and “1” for either of the second 2 options).	[30]

CES-D, Centre for Epidemiologic Studies-Depression; CIDI, Composite International Diagnostic Interview; DAWLY, disability-adjusted working life years; DSM-IV, Diagnostic and Statistical Manual of Mental Disorders, fourth edition; EHF, Eye, Hand, and Feet; GHQ-30, General Health Questionnaire-30; LF, lymphatic filariasis; MINI-Plus, Mini-International Neuropsychiatric Interview-Plus; NA, Not applicable; PHQ-9, Patient Health Questionnaire-9; SALSA, Screening Activity Limitation and Safety Awareness; SRQ-20, Self-Reporting Questionnaire-20; WHODAS, WHO Disability Assessment Schedule; WHO-DG, WHO disability grade; WHOQOL, WHO quality of life.

### Disability, psychosocial, and mental health status due to leprosy, podoconiosis, or lymphatic filariasis

#### Prevalence of leprosy-related disability

A study in Brazil included 84 patients with leprosy and found 81% with multibacillary lesions. Less than half of the patients (41.7%) had no disability at the time of the study, although 36.9% had not been evaluated for disability [31]. Another study, this time from India, showed high rates of disability with 147 (86%) of the patients having grade 2 (visible deformity) and 4 (2.3%) grade 1 disability [32]. A study in Mexico among 223 study participants affected by leprosy reported that disabilities, as assessed by WHO-Disability Grade (DG) and Eye, Hand, and Feet (EHF) score, affected 32% of participants [33,34]. Another study in 104 people affected by leprosy in Brazil found 20 (19.2%) patients with grade 2 leprosy-related deformities [34]. Similarly, a study in Ethiopia among 513 people affected by leprosy showed that 65.9% had disability, 40.2% had grade I disability, and 25.7% had grade II disability [35]. A study conducted in Indonesia among 1,358 leprosy-affected individuals showed that most impairment was associated with the feet (47%), followed by hands (31%) and eyes (11%) [36].

In contrast with the above studies, a study in Ethiopia reported a lower prevalence of grade 2 disability among new cases, with the proportion of grade II disability being 3.9%. This prevalence was lower than the national average, which was 10% [37]. A cross-sectional study in Brazil used EHF score in 282 leprosy-affected individuals and reported the maximum degree of physical incapacity (12 points) in only one case. The others presented from 0 to 8 points, and 11.3% people presented at least 2 compromised segments (which is considered a disability) [38]. A group of researchers in Brazil used the Screening Activity Limitation and Safety Awareness (SALSA) scale to measure activity limitation among 84 leprosy-affected individuals. More than half of the participants (53.6%) did not have any activity limitations, 32.1% had mild limitations, 10.7% had moderate limitation, 3.4% had severe limitation, and none of the participants had developed very severe limitations [31].

Another study in Brazil showed a mean SALSA score of 4.8 points (SD = 7.84), with scores equal to or higher than 25 points in 84 (29.8%) people [38]. The very severe limitation score was identified in 5 (1.8%) people. However, among people with limitations, the mild form was most prevalent, with 68 (24.0%) cases [38]. A study from Mexico reported that 57.8% of people affected by leprosy had some limitations in activities as assessed by the SALSA scale, with most (39%) being slight limitations [39]. A study from Brazil among people affected by leprosy reported a median SALSA score of 31.0 (25.0 to 41.5), with 24% having no significant functional activity limitations (FALs), 50% mild, 8.7% moderate, 5.8% severe, and 11.5% very severe FALs [34]. Another study conducted in Mexico assessed social participation among leprosy-affected individuals. Among the cases, 35.4% presented with some degree of restriction in social participation, with a median score of 8 [33].

#### Severity of leprosy-related disability and associated factors

A study from India found that education, occupational status, income, and duration since diagnosis had statistically significant associations (*p* < 0.05) with disability [32]. After controlling for the effect of other variables, not having an education, a longer duration of the disease, and having experienced surgery were significantly associated with disability [32]. Patients with duration of symptoms of 6 to 12 months and greater than 24 months were more likely to develop disability: adjusted odds ratio (AOR) 2.13 (CI 1.14 to 3.96), *p* = 0.017 and >24 months AOR 2.491 (CI 1.31 to 4.72), *p* = 0.005. Signs of nerve damage and reversal reactions were also associated with higher disability rates: AOR 13.1 CI (8.07 to 21.25), *p* < 0.001, and 1.85 (CI 1.03 to 3.33), *p* = 0.038, respectively [35]. A univariate analysis showed significant correlation between WHO-DG and the SALSA scale score (*p*-value 0.001) [39].

The SALSA scale was used to categorize outcomes into absence (score <25) or presence (score ≥25) of activity limitations. Using multiple logistic regression, there were significantly more activity limitations for women, for people with low income, and for people who reported pain. People who reported lesions that they considered to be significant also had greater limitations, as did having a physical disability as classified by WHO-DG [39]. There was an association between the presence of disabilities and FALs (*p* = 0.001) [34]. Increasing SALSA scores were also associated with decreasing quality of life, in terms of the physical (*r* = −0.68; *p* < 0.001), psychological (*r* = −0.28; *p* = 0.003), social (*r* = −0.21; *p* = 0.03), and environmental (*r* = −0.47; *p* < 0.001) domains of the WHOQoL-BREF [34]. Impairment status did not change significantly during treatment. Before treatment with standard MDT, 31% of people already had grade 1 impairment, and 31% had grade 2 impairment [36]. At release from treatment (RFT), 27% had grade 1, and 32% had grade 2 impairments. However, 2 to 5 years after RFT, 26% of the participants had grade 1 impairment, and 49% had grade 2 impairment. This difference was statistically highly significant [36].

Another study reported the association between participation and activity limitation among people affected by leprosy. The mean participation score was 24.4 points (SD = 7.8), ranging from 16 to 68 points. The maximum SALSA score is 60 to 80 points, which corresponds to very severe limitation. Among the cases reporting restriction of participation, mild restriction was common. Restriction of participation was significantly associated with activity limitation (*p* < 0.0001) [38]. In the univariate analysis, any reported poor physical and mental health was associated with social restriction (*p* = 0.001 in both cases). There was less restriction of social participation among people who did not present with disabilities (*p*-value = 0·001). Severe activity limitations and/or the highest level of anticipated stigma was associated with a much higher level of participation restriction (13.8 and 20.8 points, respectively) [33].

When SALSA scale scores were examined by degrees of disability, a paradox emerged with some patients with grade 0 physical disability having mild or moderate activity limitation scores. Equally, some patients with grade 2 physical disability had activity scores in the “without limitations” or “mild limitations” categories [31]. This might be explained as follows: As WHO DG measures only on leprosy-related disability, individuals having other possible impairments such as old age might contribute to the disability. Patients with leprosy reactions were 7 times more likely to develop activity limitations than those without reactions. Patients who developed physical disability were 4 times more likely to develop limitations in activities of daily living than those who had no disability [31].

The disability-adjusted life year (DALY) concept has been adapted to estimate the number of productive years lost due to disability, called disability-adjusted productive work years lost or disability-adjusted working life years (DAWLYs). A study in India among leprosy-affected individuals found the overall mean DAWLY (±SE) to be 28.6 (±0.67), indicating a significant (*p* < 0.05) reduction of 13.4 years or 31.9% from the ideal productive period of 42 years [19]. The EHF score was used to assess leprosy-related disability in a study conducted in Ethiopia. Disability as measured by EHF score was significantly positively associated with mental distress [40].

#### Psychosocial and mental health status due to leprosy

A comparative cross-sectional study of leprosy patients and healthy controls in Bangladesh assessed quality of life and reported significantly lower scores among leprosy patients for both genders (*p* = 0.01). Factors associated with decreased quality of life were the presence of perceived stigma, fewer years of education, the presence of deformities, and a lower annual income [41].

A study from India assessed mental health outcomes of leprosy and found that psychiatric disturbances tended to increase with the duration of leprosy, although the trend did not reach statistical significance. Psychiatric disturbance was more common in patients with physical deformity, and this was statistically significant. The prevalence rate of psychiatric disturbance among leprosy patients was about 9.9%. This prevalence rate was much greater than in the general population in the area in which the study was conducted [42].

A case–control study conducted among people affected by leprosy, patients with tinea versicolor, and healthy controls in Nigeria used the General Health Questionnaire (GHQ) score to evaluate mental health outcomes. The mean GHQ scores were significantly higher in the leprosy group than in the 2 control groups. The analysis of variance (ANOVA) for the 3 groups mean showed a statistically significant difference (ANOVA 19.83, *p* < 0.001) in psychiatric morbidity [43]. A psychiatric diagnosis was more commonly made among people affected by leprosy (58%) compared to those with tinea versicolor (18.2%) or healthy controls (14.8%) [43]. The most commonly reported psychiatric disorders were depressive disorder and anxiety disorder.

A cross-sectional comparative study conducted in Ethiopia among people affected by leprosy assessed the association between leprosy and mental distress using the Self-Reporting Questionnaire-20 (SRQ-20) scale. The overall prevalence of mental distress in the study population was 34.6%. Among people with leprosy, the prevalence was 52.4%, compared to 7.9% in the nonleprosy patients. After controlling for other sociodemographic variables, people with leprosy had a 7.14-fold higher risk of mental distress than nonleprosy patients [40]. When the level of disability increased, the risk of mental distress also seemed to increase. Overall, 18.5% of people affected by leprosy had suicidal ideation, while only 6.3% of the nonleprosy patients reported such thoughts in the previous month. However, the investigators thought leprosy patients might overreport symptoms of mental distress as a way of seeking attention from healthcare providers [40]. Another study, this time from Bangladesh, used the same mental health scale and found similar results. Moreover, the SRQ scores were highly correlated with total quality of life scores and physical and psychological domain scores [44].

The Centre for Epidemiologic Studies for Depression (CES-D) scale was used to assess the association between leprosy and depression in Bangladesh. The median CES-D score for leprosy patients was 28.0, while that of the control group was 12.0 (*p* < 0.001). As disability grade advanced, the total CES-D score also increased [26].

Another study assessed mental health outcomes of people affected by leprosy in Brazil using the MINI-Plus. Among 120 study participants, 71.7% had at least 1 psychiatric diagnosis. Of those with at least 1 diagnosis (86 patients), 20.8% fulfilled the criteria for 1 diagnosis, 21.7% had 2 diagnoses, and the remaining 29.2% had 3 or more psychiatric diagnoses. The diagnosis of major depressive disorder was the most common. Of all patients, 37 (30.8%) were diagnosed with current depression and 39 (32.5%) had depression in the past [28].

#### Disability due to podoconiosis

A comparative cross-sectional study among podoconiosis patients and healthy neighborhood controls in Ethiopia reported that the median WHO Disability Assessment Schedule II (WHODAS II) score was significantly higher in podoconiosis cases compared to their healthy neighbors. The mean number of days in the past 30 days in which individuals were totally unable to carry out usual activities or unable to work because of any health condition was 3.1 (±4.3) in the podoconiosis group and 0.2 (±1.1) in the healthy neighbor group (*p* < 0.001) [45]. Having acute adenolymphangioadenitis (ALA) in the past month had a statistically significant effect on depression score (*p* = 0.002). However, stage of disease did not have a significant impact on the depression score [45].

#### Psychosocial and mental health status due to podoconiosis

In another study from Ethiopia, people with podoconiosis had significantly lower mean overall quality of life scores than healthy controls, with a mean difference of −12.35 (95% CI, −13.87 to −10.83). This was also seen in all 4 subdomains (physical, psychological, social, and environmental, *p* < 0.001) [46]. Factors associated with below average quality of life scores included experiencing high levels of stigma (odds ratio (OR) = 3.71, 95% CI: 2.19 to 6.27), being illiterate (OR = 2.07, 95% CI: 1.36 to 3.21), having additional comorbidities (OR = 2.12, 95% CI: 1.19 to 4.06), and being unmarried [46].

The study described under “disability outcomes” above showed depressive symptoms to be significantly more common among people with podoconiosis (34/269, 12.6%) than their healthy neighbors (2/268 = 0.7%, *p*-value <0.001). Among participants with podoconiosis, 5.2% were considered at high risk of suicide, whereas only 0.4% among of their healthy neighbors were (*p* < 0.001) [45]. In the multivariable logistic regression model, people with podoconiosis had 11.4 times higher odds of having an elevated depressive symptom score than people without podoconiosis (95% CI 2.4 to 53.4) [45].

Another group of researchers examined mental distress among people with podoconiosis in northern Ethiopia. The mean K10 score was 15.92 (95% CI: 15.27 to 16.57) in people with podoconiosis and 14.49 (95% CI: 13.85 to 15.12) in healthy neighborhood controls (average K10 scores 1.43 points higher [95% CI: 0.52 to 2.34]). Although not linear, there was a significant difference (*p* = 0.001) in the mean K10 scores across podoconiosis disease stages [27].

Depressive symptoms measured using the Patient Health Questionnaire-9 (PHQ-9) cutoff 5 were common among people with podoconiosis and lower limb lymphoedema of other cause in Cameroon. More than one-third of participants (38.5%) presented with at least some degree of depressive symptoms, although the large majority of these were classified as having mild depression [47]. There were no significant differences in levels of depressive symptoms between people with podoconiosis (mean = 3.38, SD = 3.5) and those with lower limb lymphoedema of other cause (mean = 3.65, SD = 2.82) (*p* = 0.73) [47].

#### Disability due to lymphatic filariasis

Researchers used an 8-domain 5-level score (score ranging from 1 = no problem to 5 = extreme problem) in LF lymphoedema cases in Malawi. The majority of participants (60%) reported that they had no problem (score = 1). Approximately half of participants (51%) stated that they needed some form of assistance with their self-care, although this was mostly when they were facing acute adenolymphangitis attacks [48]. The mean overall disability score using this newly developed scale among lymphoedema cases was 13.9 with a range of 8 to 34. Pearson correlation coefficient analyses showed a significant negative association (*p* < 0.01) between overall disability score and the maximum distance participants were able to walk (*r* = −0.436; *p* < 0.001) and the hours they were able to work in an average day (*r* = −0.388; *p* < 0.001) [48].

A study of 372 people with LF in south India described several functional outcomes. About 31% of the interviewed patients and 35% of the control group felt that filariasis definitely or possibly hindered an affected person from doing domestic tasks, which included cooking, washing, cleaning, and preparing children for school [49]. During the quantitative interviews, about 28% of the patients reported altered activity, and 5% gave up their work. All activities were significantly more affected in patients with acute adenolymphangitis than in other groups [49].

#### Psychosocial and mental health status due to lymphatic filariasis

A comparative cross-sectional study of people with filariasis lymphedema and healthy controls in Sri Lanka measured quality of life using the Short-Form (SF-36). Patients experienced poorer physical functioning, more role limitations as a result of physical health problems, less emotional well-being, poorer social functioning, and more pain than healthy controls [30]. There was no association between any of the domains of the SF-36 and the number of acute adenolymphangitis attacks suffered during the past 1 year, the total number of acute adenolymphangitis attacks suffered during the entire duration of disease, or the maximum duration of lymphoedema among patients [30]. However, in the general health domain of the SF-36, cases unexpectedly reported a better general health status compared to controls [30].

This study also measured the mental health condition of the 2 groups using the General Health Questionnaire-30 (GHQ-30). The GHQ-30 score demonstrated mental well-being in 67.2% of controls, which was significantly better than that of patients (36.2%, *p* <0.001) [30]. Among patients, there was no association between GHQ-30 score and suffering at least 1 acute adenolymphangitis attack during the entire duration of the disease, the maximum grade of lymphedema, the total number of acute adenolymphangitis attacks suffered during the entire duration of the disease or duration of lymphoedema, or the maximum grade of lymphoedema (*p* > 0.05) [30].

A cross-sectional study from Nigeria examined the association between LF and depression. Among study cases, 19 (20%) met the criteria for depression, using the Composite International Diagnostic Interview (CIDI), with the severity of depression being mild in 42.1%, moderate in 31.6%, and severe in 26.3%. The percentage of those found to be depressed among people with LF (20%) was higher than the reported prevalence of depression among adults in the general population in Nigeria (3.1% to 5.2%) [50]. Logistic regression analysis revealed that history of mental illness (OR 40.8, *p* = 0.008), duration of the illness between 11 and 20 years (OR 5.0, *p* = 0.079), unemployment (OR 12.7, *p* = 0.003), and low self-esteem (OR 0.09, *p* = 0.004) were predictive of depression in the cohort [50].

## Discussion

Leprosy, podoconiosis, and LF are diseases of poverty associated with high level of disability. As well, there was high level of psychosocial and mental health impairment. This was shown in diverse studies from Africa, Asia, and South America. The 3 NTDs on which this review focuses all affect the lower limb resulting in progressive swelling or lymphoedema, deformity, and potential disability. In addition to these lower limb changes, leprosy also affects the skin, peripheral nerves, eyes, and hands. In the case of LF, in addition to lower limb changes, the upper limb, breast, or scrotum may also be affected, leading to lymphoedema or hydrocele. However, podoconiosis is limited to the lower limb leading to progressive swelling or lymphoedema.

Among the studies included in this review, the majority (*n* = 16) dealt with leprosy, and an equal number of articles (*n* = 4 each) addressed podoconiosis and LF. Concerning the quality of the included studies, aims were clearly stated in all included papers, the design was appropriate to the study in 92% of studies, evidence was provided of reliability or validity of measures in 71%, and statistics were accurately reported in 83% of the studies. However, sample selection was relatively unbiased in only 63% of the studies, and justification for sample size was given in only 50% of studies.

We were unable to perform a meta-analysis, because the studies included in this review were highly heterogeneous and used different outcome measures. Another limitation is that we only reviewed articles written in English. However, one strength is that most of the outcome measures are robust instruments with a long history of use both in studies and in clinical practice.

A range of degrees of disabilities secondary to the reviewed NTDs were reported in 14 studies [19,31–40,45,48,49]. Ten studies reported a significant association between being affected by the selected NTDs and psychosocial and mental health outcomes [26–28,40,42–45,47,50].

The prevalence of grade 2 disability varied from 3.9% in Ethiopia [37] to 86% in India [32], although a study in Ethiopia in the same calendar year reported grade 2 disability of 25.7% [35]. The reason for this discrepancy could be that the study in Ethiopia was conducted among new cases of leprosy, whereas the study in India was conducted among leprosy-affected individuals who had longer duration of disease. Longer duration of the disease is associated with severity of disability [32,35]. The authors also suggested that an aggressive control program using early diagnosis, early treatment, and full integration of the previously vertical program might have resulted in lower grade 2 disability [37]. It is worth highlighting that the 3.9% grade II disability is well below the national average, which was 10% [37].

An interesting study by Rao and colleagues reported the extent of the loss of productivity among people affected by leprosy who experience on average a loss of one-third of their productive years [19]. Leprosy is significantly associated with activity limitation as measured by the SALSA scale [31,34,36,38,39]. Physical disability is also significantly associated with activity limitation [31–33]. The severity of activity limitation and high levels of anticipated stigma were significantly associated with reduced participation [36,38]. Increased activity limitation was associated with decreased quality of life [34]. An increase in the level of disability also increased the risk of depression [26] and mental distress [40]. This is to be expected among people affected by such a chronic and disabling disease [40]. Podoconiosis patients had high disability score as measured by WHODAS II. The mean number of days in which the podoconiosis cases were unable to do their routine activities or unable to work was 3.1 days per month [45]. In the case of leprosy, there was nearly 33% loss of productivity, whereas in the case of podoconiosis, the loss was about 10%. Although different tools were used to measure productivity, it appears that the loss of productivity due to leprosy-related disability is much higher than that in podoconiosis. A study among LF patients showed that 28% of the patients reported altered activity, and 5% gave up their work completely [49].

Among the studies included, 4 revealed the effect of NTDs on quality of life or general health status. In 3 of them, quality of life was reduced significantly [34,44,46]. In contrast, one study reported that people affected by LF had a better general health status than apparently healthy controls [30]. It is possible that the selection of controls was responsible for this finding—the apparently healthy individuals who accompanied the filarial patients may have been long-term burned-out caregivers. This could also be explained by the disability paradox, as surveys have shown that people with disabilities report a quality of life that is either as good as or even better than that of nondisabled people [51].

Several mental disorders are associated with NTDs ranging from mild panic disorders to generalized anxiety and major depressive disorders. A significant association between leprosy and mental disorders was reported in 6 articles [26,28,40,42–44]. There was also a significant association between mental disorders and podoconiosis [27,45,47] and between mental disorders and LF [30,50]. The risk of suicide was significantly higher in study participants with podoconiosis (5.2%) than their healthy neighbors (0.4%) [45]. The suicidal ideation was also higher among leprosy patients: 18.5% of people affected by leprosy had suicidal ideation as compared to only 6.3% patients affected by other skin diseases [40].

A recent systematic review assessed the impact of leprosy on mental well-being, which found that leprosy-affected individuals are at risk of poor psychosocial and mental health outcomes. The reported psychosocial outcomes were fear, shame, low self-esteem, loneliness, sadness, anger, and low quality of life [52]. |Several mental health conditions were reported in the review, such as depression, anxiety disorders, mental distress, and suicide (thoughts and attempts). The most commonly reported mental health condition was depression, and the second most common was anxiety disorder [52].

The mental health outome most often reported by studies was depression [26,28,43,45,47,50], with mental distress—which is a state of poor mental well-being that can involve a range of different symptoms, including symptoms of depression and anxiety—second [27,28,40,43]. This suggests the importance of provision of access to mental health screening and of appropriate mental health interventions to people affected by these 3 NTDs [50].

In conclusion, the NTDs described in this study all result in important disability, psychosocial, and mental health impairments. In response to this finding, any recommended intervention for podoconiosis, LF, and leprosy should be holistic, integrating physical care with psychosocial and mental healthcare.

**Key learning points**

- There is extensive physical disability due to leprosy, podoconiosis, and lymphatic filariasis (LF).
- Poor quality of life is common among people affected by each of these 3 neglected tropical diseases (NTDs).
- Reduced mental well-being (mainly depression and mental distress) is common among people affected by leprosy, podoconiosis, and LF.
- To prevent long-term disability due to leprosy, podoconiosis, and LF, early case finding and early management of cases are needed.
- Integrated physical, psychosocial, and mental health interventions are vital for the management of leprosy, podoconiosis, and LF.

**Top five papers**

1. Bartlett J, Deribe K, Tamiru A, Amberbir T, Medhin G, Malik M, et al. Depression and disability in people with podoconiosis: a comparative cross-sectional study in rural Northern Ethiopia. Int Health. 2016;8(2):124–31.
2. Rocha-Leite CI, Borges-Oliveira R, Araújo-de-Freitas L, Machado PRL, Quarantini LC. Mental disorders in leprosy: An underdiagnosed and untreated population. J Psychosom Res. 2014;76(5):422–5.
3. Rao PS, Darlong F, Timothy M, Kumar S, Abraham S, Kurian R. Disability adjusted working life years (DAWLYs) of leprosy affected persons in India. Indian J Med Res. 2013;137(5):907–10.
4. Van Brakel WH, Sihombing B, Djarir H, Beise K, Kusumawardhani L, Yulihane R, et al. Disability in people affected by leprosy: the role of impairment, activity, social participation, stigma and discrimination. Glob Health Action. 2012;5.
5. Mousley E, Deribe K, Tamiru A, Davey G. The impact of podoconiosis on quality of life in Northern Ethiopia. Health Qual Life Outcomes. 2013;11:122.

## Supporting information

S1 Text Protocol for systematic review. (PDF)
