# Supplementary material for: The impact of podoconiosis, lymphatic filariasis, and leprosy on disability and mental well-being: A systematic review
Source: PLoS Negl Trop Dis. 2021 Jul 8;15(7):e0009492. doi: 10.1371/journal.pntd.0009492 (PMC8266075; doi:10.1371/journal.pntd.0009492)
Supplement: S1 Text — (PDF) [file pntd.0009492.s001.pdf]

NHS
National Institute for
Health Research

Psychosocial and functional (disability) outcome of podoconiosis, lymphatic filariasis and leprosy

Oumer Ali Ahmed, Hattie Sharp, Maya Semrau, Abraham Tesfaye, Asrat Mengiste, Abebaw Fekadu, Gail Davey

Citation

Oumer Ali Ahmed, Hattie Sharp, Maya Semrau, Abraham Tesfaye, Asrat Mengiste, Abebaw Fekadu, Gail Davey. Psychosocial and functional (disability) outcome of podoconiosis, lymphatic filariasis and leprosy. PROSPERO 2019 CRD42019128400 Available from:
http://www.crd.york.ac.uk/PROSPERO/display_record.php?ID=CRD42019128400

Review question

1. What are the functional/disablity outcomes secondary to LF, podoconiosis and leprosy

2. What are the psychosocial outcomes secondary to LF, podoconiosis and leprosy?

Searches

Search Engines: PubMed/MEDLINE, COCHRANE, GLOBAL HEALTH database, PsycINFO and EMBASE: The search strategy will be developed in PubMed, and iterative changes will be made to enable optimal search in the other databases.

There is no time restriction as we didn’t come across previous review on the same topic. However, there is language restriction (English only), and restriction on studies conducted only in Endemic countries.

The search terms includes - Podoconiosis OR elephantiasis OR leprosy, Disability OR function OR "mental distress" OR depress* OR alcohol OR psychosocial OR Substance OR “Anxiety disorder” OR “common mental disorder” OR “Mood disorder”.

On the search term I have added list of endemic countries for podoconiosis, lymphatic filariasis and leprosy. I got the list from World Health Organisation (WHO) website

Search strategy

Types of study to be included

Cross-sectional studies, prospective studies, case-control studies, clinical trials

Condition or domain being studied

Condition: Disablity (functional outcome), mental distress, depression, substance abuse/alcohol (psychosocial outcomes) secondary to LF, podoconiosis and leprosy.

Participants/population

People with podoconiosis, lymphatic filariasis and leprosy; any age, any gender and any severity with clearly stated disability and psychosocial outcomes. Studies on these NTDs that do not address disability or co-morbid psychosocial disorders will be excluded.

Intervention(s), exposure(s)

Main outcome will be disability and psychosocial outcomes secondary to LF, podoconiosis and leprosy with or without any interventions intended to minimize these outcomes. However, the impact of the intervention will be evaluated separately.

Comparator(s)/control

Not relevant

Context

The focus of the study will be on neglected tropical diseases, specifically LF, podoconiosis. The reason for focusing on theses diseases is they are a group of chronic, disabling, and disfiguring conditions that occur most commonly in the setting of extreme poverty, especially among the rural poor and some disadvantaged urban populations. Despite the substantial disease burden they impose, NTDs have largely been ignored in the global health architecture until recently. We want to understand the gap on disability and psychosocial impacts so that appropriate intervention will be recommended

Main outcome(s)

The main outcomes are disability and depression. The disability will be measured using the World Health Organisation Disability Assessment Schedule (WHODAS II) , which is a validated disability tool.

WHODAS 2.0 assesses the followings:- standing for long periods such as 30 minutes, taking care of household responsibilities, learning a new task, how much of a problem for joining community activities in the same way as anyone else can, emotional affection by health problems, concentrating for 10 minutes on doing something, walking a long distance such as a kilometre or equivalent, getting dressed, maintaining a friendship and maintaining day-to-day work.

The other main outcome is depression measured by Patient Health questioner (PHQ-9), a validated mental health assessment tool.

Each question requires participants to rate the frequency of a depressive symptom experienced in the two weeks prior to evaluation. These : 1.Little interest or pleasure in doing things 2.depressed mood, 3.insomnia or hypersomnia, 4.fatigue or loss of energy, 5.appetite disturbances, 6.guilt or worthlessness, 7. diminished ability to think or concentrate, 8. psychomotor agitation or retardation, and 9.suicidal thoughts. Scores range from 0 (“not at all”) to 3 “nearly every day” with a total score ranging from 0 to 27 .

Timing and effect measures

Not applicable

Additional outcome(s)

The secondary outcomes are mental distress/anxiety and alcohol use disorder. Mental distress will be measure by Hamilton Anxiety rating scale (HAM-A) , alcohol use disorder will be measured by FAST.

Timing and effect measures

Not applicable

Data extraction (selection and coding)

a) Authors OA and HS will do the database search and the manual search of the reference lists with additional support from MS and AT

b) OA and HS will screen identified citations independently according to the selection criteria including rapid appraisal of full manuscripts. If no consensus is reached between OA and HS, then MS and AT will review.

c) Excluded articles and reasons for exclusion will be documented.

Risk of bias (quality) assessment

ProProtocol will define the method of literature critique/ appraisal use, and will use PRISMA tool for relevant con content and methodology used in the each of the papers to be reviewed

We will check the followings critically: aims clearly stated, design appropriate to stated objectives, justification for sample size, evidence provided of reliability or validity of measures used and statistics accurately reported.

Strategy for data synthesis

Narrative synthesis will be done using a framework which consists of four elements

1. Assessing the psychosocial and disability outcomes of the 3 NTDS

2. Exploring relationships among studies

3. Assessing the interrelatedness of disabilities among these NTDs

4. Assessing the strength of the synthesis

Analysis of subgroups or subsets
Analysis will be stratified by the three disease entities

Contact details for further information
Oumer Ali Ahmed
O.A.Ahmed@bsms.ac.uk

Organisational affiliation of the review
CDT Africa- Addis Ababa University, Brighton and Sussex Medical School

Review team members and their organisational affiliations
Dr Oumer Ali Ahmed. CDT Africa- Addis Ababa University, Brighton and Sussex Medical School
Ms Hattie Sharp. Brighton and Sussex Medical School
Dr Maya Semrau. Brighton and Sussex Medical School
Dr Abraham Tesfaye. Addis Ababa University
Dr Asrat Mengiste. Addis Ababa University
Professor Abebaw Fekadu. Addis Ababa University, Brighton and Sussex Medical School
Professor Gail Davey. Brighton and Sussex Medical School

Type and method of review
Epidemiologic, Narrative synthesis, Systematic review

Anticipated or actual start date
21 January 2019

Anticipated completion date
20 June 2019

Funding sources/sponsors
The review is part of Excellence in Disability Prevention Integrated across Neglected Tropical Diseases' (EnDPoINT) project work-package 1. The study is supported by National Institute of Health Research. The Funders do not have role in commissioning or advising in the review

Conflicts of interest

Language
English

Country
England, Ethiopia

Published protocol

Stage of review
Review Ongoing

Subject index terms status

Subject indexing assigned by CRD

Subject index terms
Elephantiasis; Elephantiasis, Filarial; Humans; Leprosy

Date of registration in PROSPERO
30 July 2019

Date of publication of this version
30 July 2019

Details of any existing review of the same topic by the same authors

Stage of review at time of this submission

Stage	Started	Completed
Preliminary searches	Yes	No
Piloting of the study selection process	No	No
Formal screening of search results against eligibility criteria	No	No
Data extraction	No	No
Risk of bias (quality) assessment	No	No
Data analysis	No	No

Versions
30 July 2019

PROSPERO
This information has been provided by the named contact for this review. CRD has accepted this information in good faith and registered the review in PROSPERO. The registrant confirms that the information supplied for this submission is accurate and complete. CRD bears no responsibility or liability for the content of this registration record, any associated files or external websites.
